# Manipulating the Light Systemic Signal HY5 Greatly Improve Fruit Quality in Tomato

**DOI:** 10.1002/advs.202500110

**Published:** 2025-04-11

**Authors:** Jiachun Wang, Xiaomeng Li, Jiajia Li, Han Dong, Zhangjian Hu, Xiaojian Xia, Jingquan Yu, Yanhong Zhou

**Affiliations:** ^1^ Department of Horticulture Zijingang Campus Zhejiang University 866 Yuhangtang Road Hangzhou 310058 P.R. China; ^2^ College of Horticulture Northwest A&F University Yangling Shaanxi 712100 P.R. China; ^3^ Hainan Institute Zhejiang University Sanya 572000 China

**Keywords:** Carotenoids, Fruit ripening, Invertase, Light signaling, Solanum Lycopersicum, Transcriptional regulation

## Abstract

Fruit ripening in tomato fruits comprises dramatic metabolic changes that are regulated by environmental factors. Light not only drives photosynthesis but also acts as a critical signal regulating plant growth, development, and the quality of produce. However, it is unclear how plants sense light signals in the environment to regulate fruit quality. It is demonstrated that the accumulation of Long Hypocotyl 5 (HY5) protein peaks at the breaker stage of fruit maturity, independent of fruit bagging. Genetic manipulation of HY5 reveals that its knockout delays carotenoid synthesis and sucrose conversion, while its overexpression promotes fruit ripening. Molecular and biochemical analyses show that HY5 directly activates the transcript of the key carotenoid synthesis genes, such as *Phytoene Synthase 1 (PSY1)* and *Phytoene Desaturase (PDS)*, as well as the sucrose metabolism genes, including *Lycopersicum Invertase* (*LIN5*, *LIN6*), *Vacuolar Invertase* (*VI*) and *Sucrose Synthase* (*SS1*, *SS7*). Importantly, grafting experiments reveal that HY5 acts as a systemic signal, translocating from leaves to fruits to promote ripening. Furthermore, nightly lighting with red or blue LED greatly improves fruit quality. In summary, the results establish that HY5 as a mobile protein that mediates the systemic light regulation of fruit ripening, offering practical applications for improving fruit quality.

## Graphical abstract

This study establishes that HY5, activated by low‐dose light, acts as a systemic signal to enhance tomato fruit quality by directly promoting carotenoid synthesis and sugar metabolism. These findings demonstrate HY5's mobility from leaves to fruits and its application potential through nighttime LED lighting, offering a practical strategy for crop biofortification.

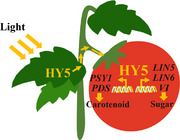

## 1 Introduction

Fruit ripening is a developmental process that involves intricate physiological and metabolic changes. Appealing colors, flavors, and volatile compounds are typical characteristics of ripe fruits.^[^ ^1^ ^]^ In tomato fruits, carotenoid synthesis and sugar metabolism are central to the ripening process. Specifically, PSY1 and PDS are rate‐limiting enzymes for the synthesis of lycopene and β‐carotene, the major carotenoids in ripe tomato fruits.^[^ ^2^, ^3^ ^]^ Concurrently, the degradation of starch during fruit maturation contributes to increased sugar content in ripe fruit, as the accumulated starch in young fruits is progressively converted into soluble sugars.^[^ ^4^ ^]^ Moreover, efficient sugar transport and accumulation are the prerequisites for fruit enlargement and flavor formation. During fruit ripening, the cell wall invertase (CWINV, encoded by *LINs*) and vacuolar invertase (VI) respectively mediate the hydrolysis of disaccharide (sucrose) to monosaccharide (fructose and glucose) in the apoplast and vacuole, which is the basis of fruit sweetness.^[^ ^5^ ^]^ Additionally, sucrose synthase (SS) enzyme regulates the bidirectional conversion between sucrose and its monosaccharide components, ensuring sugar homeostasis and contributing to the quality of fruits.^[^ ^6^ ^]^

Light affects multiple aspects of plant growth, development, and fruit quality. Besides providing energy for photosynthesis which is the source of sugar accumulation, light also acts as the signal for regulating plant growth and development. Supplemental lighting with high light intensity increases the accumulation of soluble sugars in apple and sweet pepper fruits, thereby enhancing their flavor characteristics.^[^ ^7^, ^8^ ^]^ Light induces a strong increase in anthocyanin content in strawberry (*Fragaria × ananassa*) during fruit ripening due to the upregulation of anthocyanin synthesis genes.^[^ ^9^ ^]^ Blue light enhances the ethylene‐induced degreening of citrus fruits during the postharvest stage by promoting the expression of chlorophyll catabolism genes.^[^ ^10^ ^]^ Additionally, light enhances anthocyanin accumulation in pear (*Pyrus pyrifolia*) by activating the transcription of the *MYB10* gene, thus promoting fruit coloring.^[^ ^11^ ^]^ Furthermore, far‐red light increases the partitioning of sugars to tomato fruits in association with the upregulation of genes involved in both sugar transport and metabolism, leading to higher levels of glucose and fructose in ripe fruits.^[^ ^12^ ^]^ However, the precise molecular mechanisms by which light signal regulates carotenoid and sugar metabolism during tomato fruit ripening are still not fully understood.

Phytochrome interacting factors (PIFs) and HY5 are key regulators that relay the light signals downstream of light sensing and constitute an intricate transcriptional regulation network, engaging in various facets of plant growth and development.^[^ ^13^ ^]^ When sunlight passes through the green fruit, the self‐shading by the fruit shoulder activates PIFs in the fruit end to repress the transcription of carotenoid synthesis genes.^[^ ^14^ ^]^ HY5 is well known to participate in photomorphogenesis by positively regulating the transcription of *Genomes Uncoupled 5* and *Light‐Harvesting Complex 4*, thereby promoting chlorophyll accumulation.^[^ ^15^ ^]^ By contrast, the degradation of HY5 protein curtails the expression of chlorophyll synthetic genes, leading to a reduction in chlorophyll content in tomato plants.^[^ ^16^ ^]^ Additionally, HY5 is implicated in the accumulation of anthocyanin in a light‐dependent manner.^[^ ^17^ ^]^ Furthermore, HY5 exerts influence over soluble sugar levels during plant growth and development. In tomato leaves, HY5 boosts the mRNA levels of genes associated with starch degradation, thereby contributing to the control of sugar mobilization in tomato leaves.^[^ ^18^ ^]^ Nonetheless, the precise roles of HY5 in controlling carotenoid synthesis and sugar metabolism during tomato fruit ripening remain to be characterized.

In this study, we demonstrate that HY5 is intimately involved in tomato fruit ripening through upregulating the transcripts of carotenoid synthetic and sugar metabolic genes. Molecular assays further reveal that HY5 directly transcriptionally regulates these genes. Intriguingly, grafting experiments confirm that HY5 functions as a systemic signal, transmitting from leaves to fruits to promote ripening. Additionally, nightly supplemental lighting improves the fruit quality. These findings establish HY5 as a mobile signal that integrates systemic light signaling to regulate fruit ripening, providing new insights into the regulatory mechanisms underlying fruit quality and offering practical strategies for crop improvement.

## 2 Results

### 2.1 Light Promotes the Accumulation of Carotenoids and Soluble Sugars during Tomato Fruit Ripening

**Figure 1:**
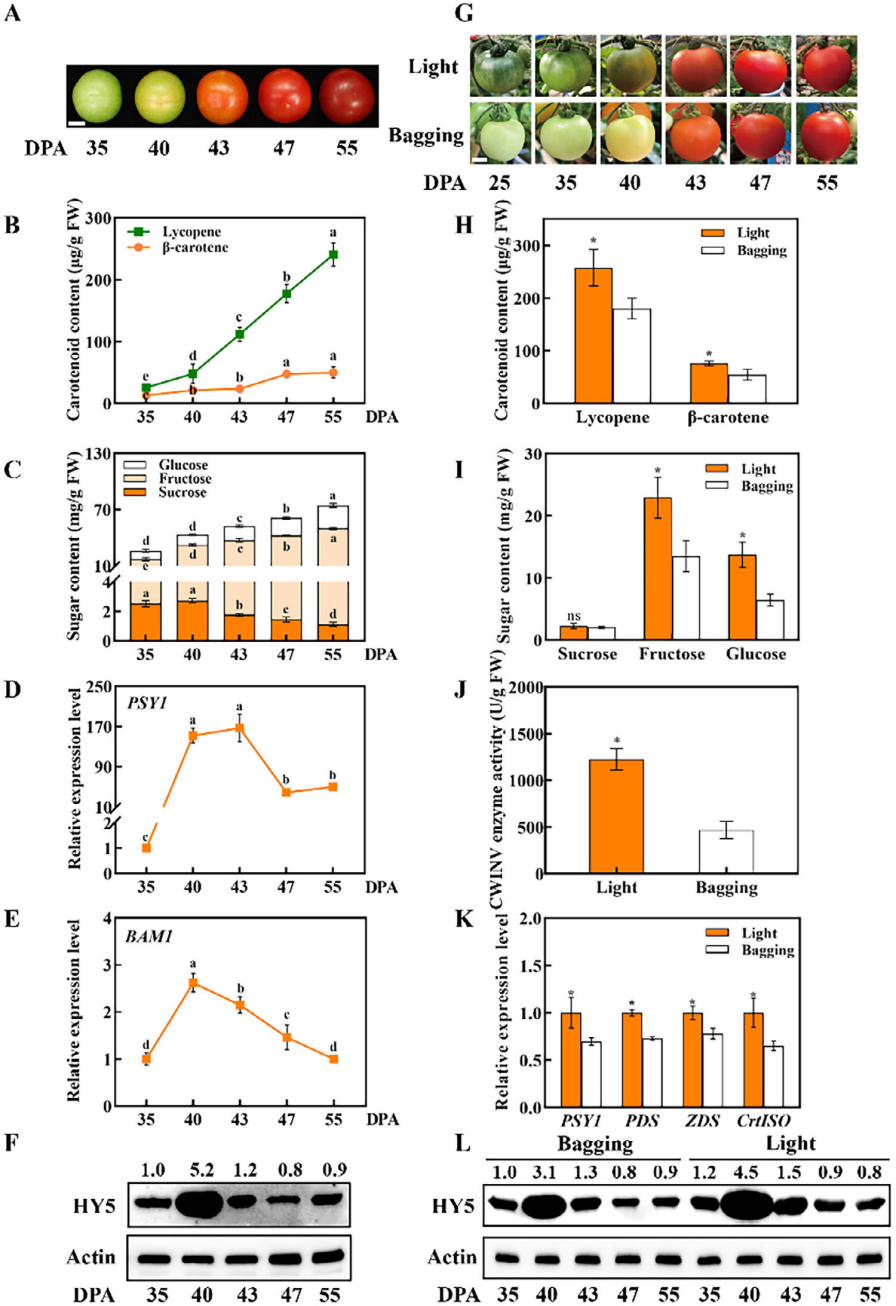
The role of light in carotenoid and sugar contents during tomato fruit ripening. A) WT fruits at 35, 40, 43, 47, and 55 DPA, respectively (Bar = 2 cm). B) Contents of lycopene and β‐carotene in WT fruit at 35, 40, 43, 47, and 55 DPA. C) Sugar (including sucrose, fructose, and glucose) contents in WT fruits at different stages during tomato ripening. D,E) qPCR analysis of *PSY1* and *BAM1* in WT fruits at 35, 40, 43, 47, and 55 DPA. F) Diurnal changes in the accumulation of HY5 protein in WT fruits at 35, 40, 43, 47, and 55 DPA. G) Representative 25, 35, 40, 43, 47, and 55 DPA fruits from light and bagging treatments (Bar = 2 cm). H) Contents of lycopene and β‐carotene in WT fruits with light and bagging treatments at 55 DPA. I) Contents of soluble sugars, including sucrose, fructose, and glucose, in WT fruits under light and bagging treatments at 55 DPA. J) The activity of CWINV in WT fruits under light and bagging treatments at 55 DPA. K) The relative mRNA levels of *PSY1* and *PDS* in WT fruits with light and bagging treatments at 43 DPA. L) Changes in the accumulation of HY5 protein with light and bagging treatment during WT fruit ripening. The data in B‐E and H‐K are presented as the mean values ± SD (*n* = 3). Different letters and asterisks indicate significant differences according to Tukey and Student's *t*‐test (*P* < 0.05). SD, standard deviation. DPA, days post anthesis; WT, wild type; CWINV, cell wall invertase; qPCR, quantitative PCR.

Throughout the process of “Ailsa Craig” tomato fruit ripening, discernible changes in surface color were observed, transitioning from yellow at 40 days post anthesis (DPA) to orange, red, and finally dark red at 43, 47, and 55 DPA, respectively (**Figure** **1**A). These shifts in coloration were accompanied by a concurrent accumulation of carotenoids. Remarkably, levels of lycopene and β‐carotene exhibited a steady increase at 40 DPA (the breaker stage, Figure 1B). In comparison, sucrose levels showed a notable decline from 43 DPA, whilst the contents of glucose and fructose exhibited an upward trend (Figure 1C). Notably, the transcript abundance of carotenoid synthesis genes [including *PSY1*, *ζ‐carotene Desaturase* (*ZDS*), *PDS*, *Carotene Isomerase* (*CrtISO*), *1‐deoxy‐d‐xylulose 5‐phosphate Synthase* (*DXS*) and *Geranylgeranyl Pyrophosphate Synthase* (*GGPS*)] and starch degradation genes [including *β‐amylases 1* (*BAM1*), *β‐amylases* (*BAM*), *Phosphoglucan Water Dikinase* (*PWD*) and *β‐amylases 8* (*BAM8*)] peaked at 40 or 43 DPA (Figure 1D,E; Figure S1, Supporting Information). Since HY5 acts as the hub of transcriptional network in response to light,^[^ ^13^ ^]^ we next measured the accumulation of HY5 protein during fruit ripening. Intriguingly, its accumulation peaked at 40 DPA, which was consistent with the trend of carotenoid and sugar contents (Figure 1F).

To study the role of local light in forming quality attributes during tomato fruit development, pigments, and sugar metabolisms were compared between fruits with or without light exposure. Tomato fruits at 15 DPA were subject to bagging using light‐impermeable papers, while the control fruits were exposed to natural light in the canopy. The natural light exposed fruits showed dark green shoulders and green color on the fruit surface, up to 35 DPA. By contrast, bagging strongly decreased the levels of chlorophyll (Chl a and Chl b), leading to a pale green color of the fruits (Figure 1G; Figure S2A,B, Supporting Information). Interestingly, fruit ripening started from 40 DPA in both control and bagged fruits (Figure 1G). However, control fruits showed a darker red color as compared to bagged fruits at the fully ripe stage (55 DPA). Accordingly, the transcript abundance of carotenoid synthesis genes (including *PSY1*, *PDS*, *ZDS*, and *CrtISO*) was decreased by bagging as compared to control (Figure 1K; Figure S2F, Supporting Information). The lycopene and β‐carotene contents in the bagged fruits were decreased by 29.8% and 24.6%, respectively, compared to control at 55 DPA (Figure 1H). Meanwhile, the levels of glucose and fructose in the bagged fruits were lower than those in control fruits at 55 DPA, whereas no significant differences in the contents of sucrose and organic acids (citric acid and malic acid) were observed (Figure 1I; Table S1, Supporting Information). Furthermore, bagging led to a significant decline in the activity of CWINV at 55 DPA and the transcript levels of genes encoding CWINV (*LIN5*, *LIN6*, and *LIN8*) at 43 DPA (Figure S2G, Supporting Information). Notably, there was a strong and transient increase in *HY5* transcripts at 40 DPA, followed by a gradual decline as fruit ripening progressed. In contrast, the transcripts of *Constitutively Photomorphogenic 1* (*COP1*), the antagonistic factor of HY5, significantly decreased at 40 DPA (Figure S3A,B, Supporting Information). Changes in HY5 protein levels mirrored that of *HY5* transcripts as the lower accumulation of HY5 protein was found in the bagged fruits at 40 DPA (Figure 1L). Therefore, the accumulation of HY5 protein correlates well with the changes in transcript abundance of genes encoding cell wall invertase. Collectively, these findings underscore that HY5 is involved in the light‐induced accumulation of carotenoids and monosaccharides of ripe fruits in tomato.

### 2.2 HY5 is Involved in the Regulation of Tomato Fruit Ripening

**Figure 2:**
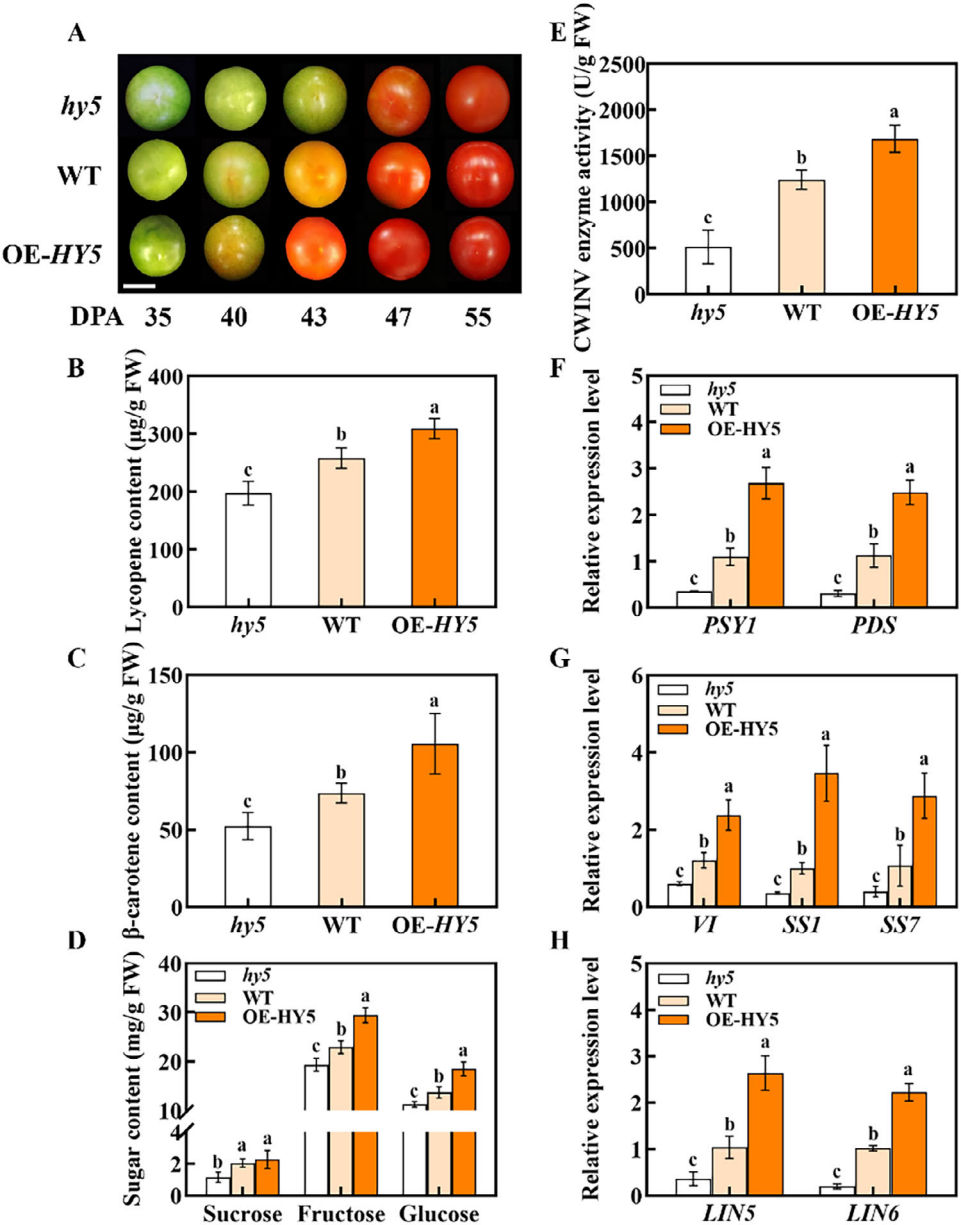
HY5 is involved in light‐regulated tomato fruit ripening. A) Fruits collected from *hy5*, WT, and OE‐*HY5* at 35, 40, 43, 47, and 55 DPA (Bar = 2 cm). B,C) Contents of lycopene and β‐carotene in the *hy5*, WT, and OE‐*HY5* fruits at 55 DPA. D) Contents of soluble sugars, including sucrose, fructose, and glucose, in the *hy5*, WT, and OE‐*HY5* fruits at 55 DPA. E) The activity of CWINV enzyme in the *hy5*, WT, and OE‐*HY5* fruits at 55 DPA. F) qPCR analysis of the carotenoid biosynthetic genes (*PSY1* and *PDS*) in the *hy5*, WT, and OE‐*HY5* fruits at 43 DPA. G) qPCR analysis of *VI*, *SS1*, and *SS7* in the *hy5*, WT, and OE‐*HY5* fruits at 43 DPA. H) qPCR analysis of *LIN5* and *LIN6* in the *hy5*, WT, and OE‐*HY5* fruits at 43 DPA. Data are presented as the means of three replicates ± SD; (*n* = 3). Different letters indicate significant differences (*P* < 0.05, Tukey's test). WT, wild type; CWINV, cell wall invertase; SD, standard deviation.

The *hy5* CRISPR mutant, WT and *HY5*‐overexpressing (OE‐*HY5*) plants were used to determine the role of HY5 in fruit maturity. In WT and OE‐*HY5* plants, fruits reached the breaker stage at 40 DPA. However, in the *hy5* CRISPR mutant, the onset of fruit ripening was delayed by 3 d (**Figure** **2**A). During ripening, the *hy5* mutant fruits showed weaker coloring than WT and OE‐*HY5*. Notably, mutation of *HY5* led to a significant decrease in the lycopene and β‐carotene levels at 55 DPA. Conversely, the overexpression of *HY5* significantly increased the carotenoid content (Figure 2B,C). The expression levels of carotenoid biosynthesis genes *PSY1*, *PDS*, *ZDS*, and *CrtISO* were increased in OE‐*HY5* fruits and decreased in *hy5* fruits (Figure 2F; Figure S5B, Supporting Information). Similarly, the levels of fructose, glucose and total soluble solids (TSS) increased in OE‐*HY5* fruits but decreased in *hy5* fruits compared to WT. However, the sucrose content exhibited subtle differences among different genotypes (Figure 2D; Figure S5A, Supporting Information). Meanwhile, the CWINV activity and the transcripts of the encoding genes were elevated in OE‐*HY5* fruits but it was attenuated in *hy5* mutant fruits compared to WT (Figure 2E,H; Figure S5C, Supporting Information).

Based on the RNA‐seq between WT and *hy5* fruits at 40 DPA, we identified 1520 differentially expressed genes, including 968 upregulated genes and 552 downregulated genes [/log_2_(fold change)/ > 1, *P*‐adj < 0.05] in *hy5* plants (Figure S4A, Supporting Information). Through analysis of kyoto encyclopedia of genes and genomes (KEGG) categories, we found the expression of genes related to starch and sucrose metabolism significantly changed in *hy5* plants (Figure S4B, Supporting Information). The crucial genes involved in starch and sugar metabolism [including *Disproportionating Enzyme 1* (*DPE1*), *Maltose excess protein 1* (*MEX1*), *VI*, *SS1* and *SS7*] were decreased in *hy5* fruits (Figure 2G; Figure S4C,D, Supporting Information). Meanwhile, RNA‐Seq data and RT‐qPCR showed that carotenoid metabolism genes (including *PSY1*, *PDS*, *ZDS*, *CrtISO*) were differentially expressed in *hy5* fruits (Figure S4F,G, Supporting Information). Taken together, the results suggest that HY5 positively regulates tomato fruit ripening by promoting carotenoid synthesis and sucrose conversion.

### 2.3 HY5 Directly Regulates the Transcription of Carotenoid Synthesis and Sugar Metabolism Genes

HY5 plays a pivotal role in the transcription of a wide array of light‐responsive genes by binding to distinct *cis*‐acting elements in the promoters, including the Z‐box (TACGTGT), C/G‐box (GACGTG), and ACGT‐containing elements.^[^ ^13^ ^]^ Examination of the promoters of carotenoid synthesis genes (*PSY1* and *PDS*), sugar metabolism genes (*VI*, *SS1* and *SS7*), and cell wall invertase genes (*LIN5* and *LIN6*) indicated that these genes contained HY5‐binding *cis*‐acting elements (Figure S5, Supporting Information) and were the potential transcriptional targets of HY5.

**Figure 3:**
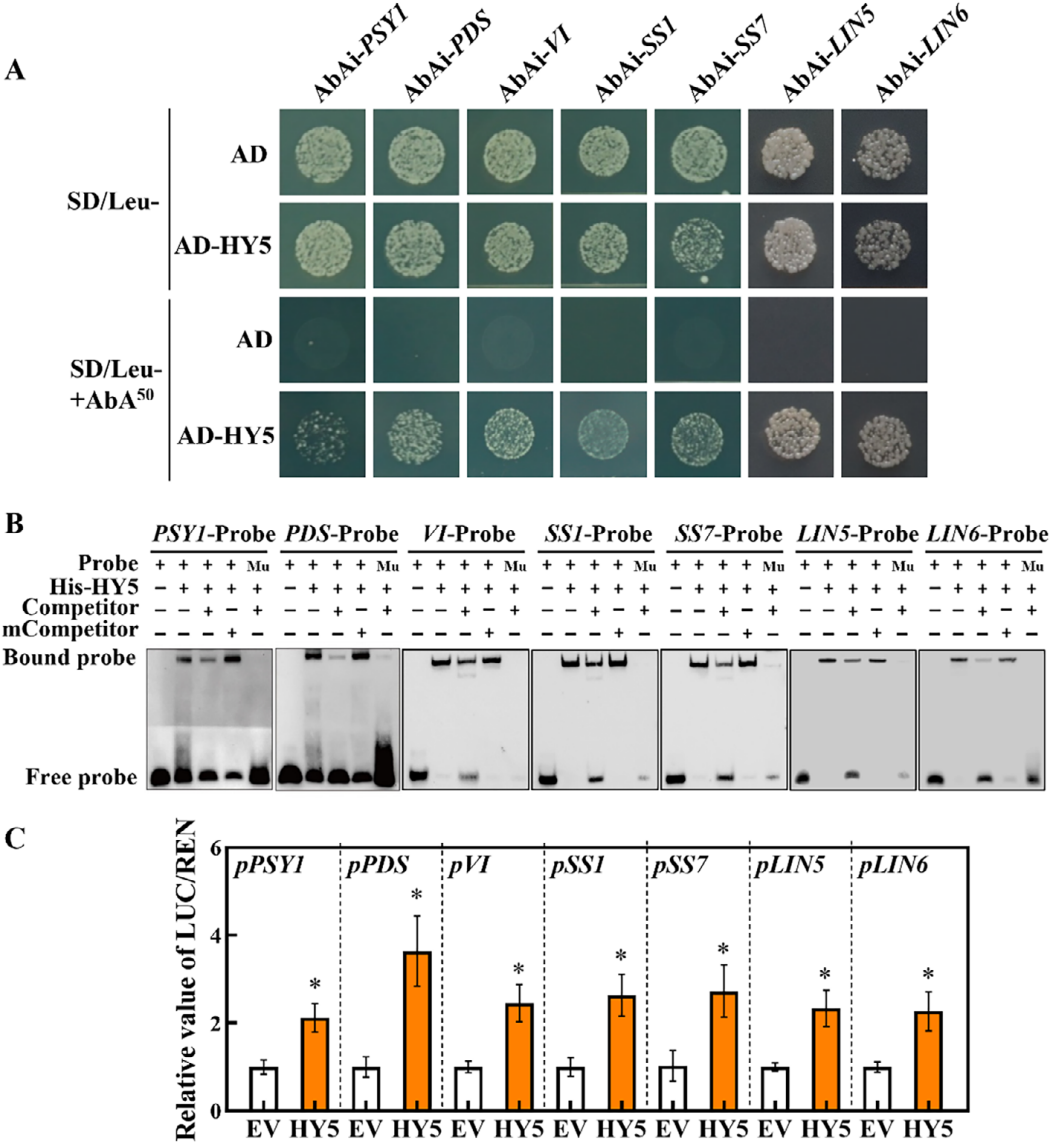
HY5 directly binds to the promoters of *PSY1*, *PDS*, *VI*, *SS1*, *SS7*, *LIN5*, and *LIN6* to participate in carotenoid accumulation and sugar metabolism during tomato fruit ripening. A) Y1H assay of HY5 binding to the promoters of *PSY1*, *PDS*, *VI*, *SS1*, *SS7*, *LIN5* and *LIN6*. B) EMSA assays showing the direct binding of HY5‐His fusion protein to HY5 *cis*‐acting elements on the promoters of *PSY1*, *PDS*, *VI*, *SS1*, *SS7*, *LIN5*, and *LIN6* in vitro. +, presence of corresponding proteins and probes. ‐, absence of the corresponding proteins and probes. C) Dual‐luciferase assays for the regulatory effect of HY5 on the expression of *PSY1*, *PDS*, *VI*, *SS1*, *SS7*, *LIN5*, and *LIN6*. The ratio of LUC/REN of the empty vector was used as the control, and its activity was taken as one. Data are presented as the means of three replicates ± SD; *n* = 4. The asterisks indicate significant differences according to Student's *t*‐test (*P* < 0.05). qPCR, quantitative PCR; Y1H, yeast one‐hybrid; EMSA, electromobility shift assay.

Compared to WT fruits, *hy5* mutant fruits exhibited a reduction, while OE‐*HY5* fruits displayed a substantial increase in the expression of *PSY1*, *PDS*, *VI*, *SS1, SS7, LIN5*, *LIN6*, and *LIN8* (Figure 2F,G). To study the direct interaction between HY5 and the promoters of *PSY1, PDS*, *VI*, *SS1, SS7, LIN5*, and *LIN6*, a yeast one‐hybrid (Y1H) assay was performed. Yeast cells carrying the pGADT7‐HY5 vector and the *P_PSY1_*‐, *P_PDS_*‐, *P_VI_*‐, *P_SS1_*‐, *P_SS7_*‐, *P_LIN5_*‐ and *P_LIN6_*‐baits were able to proliferate in SD‐Leu medium supplemented with 50 ng mL^−1^ of aureobasidin A (AbA) (**Figure** **3**A). Subsequently, electrophoretic mobility shift assays (EMSA) were employed to substantiate the formation of HY5‐DNA complex as shown by a noticeable shift in the mobility of labeled probes, indicating its binding to the HY5 *cis*‐acting elements in the promoters of *PSY1*, *PDS*, *VI*, *SS1*, *SS7*, *LIN5* and *LIN6* genes in vitro (Figure 3B). Intriguingly, these bindings were disrupted when the ACGT sequences were mutated to TTTT. Furthermore, the intensity of the shifted band was reduced in the presence of unlabeled competitor probes in a concentration‐dependent manner. Importantly, this effect was not present when unlabeled mutant probes were added (Figure 3B). Dual‐luciferase assays provided additional evidence that the activities of *P_PSY1_*‐, *P_PDS_*‐, *P_VI_*‐, *P_SS1_*‐, *P_SS7_*‐, *P_LIN5_*‐ and *P_LIN6_*‐baits were all significantly augmented by over two‐fold due to the addition of HY5 (Figure 3C). Collectively, these experiments demonstrate that HY5 directly binds to the promoters of *PSY1*, *PDS*, *VI*, *SS1, SS7, LIN5*, and *LIN6* consequently upregulating their expression and contributing to the regulation of carotenoid synthesis and sugar metabolism.

### 2.4 HY5 Functions as a Long Distal Signal to Trigger Carotenoid Synthesis and Sugar Metabolism

**Figure 4:**
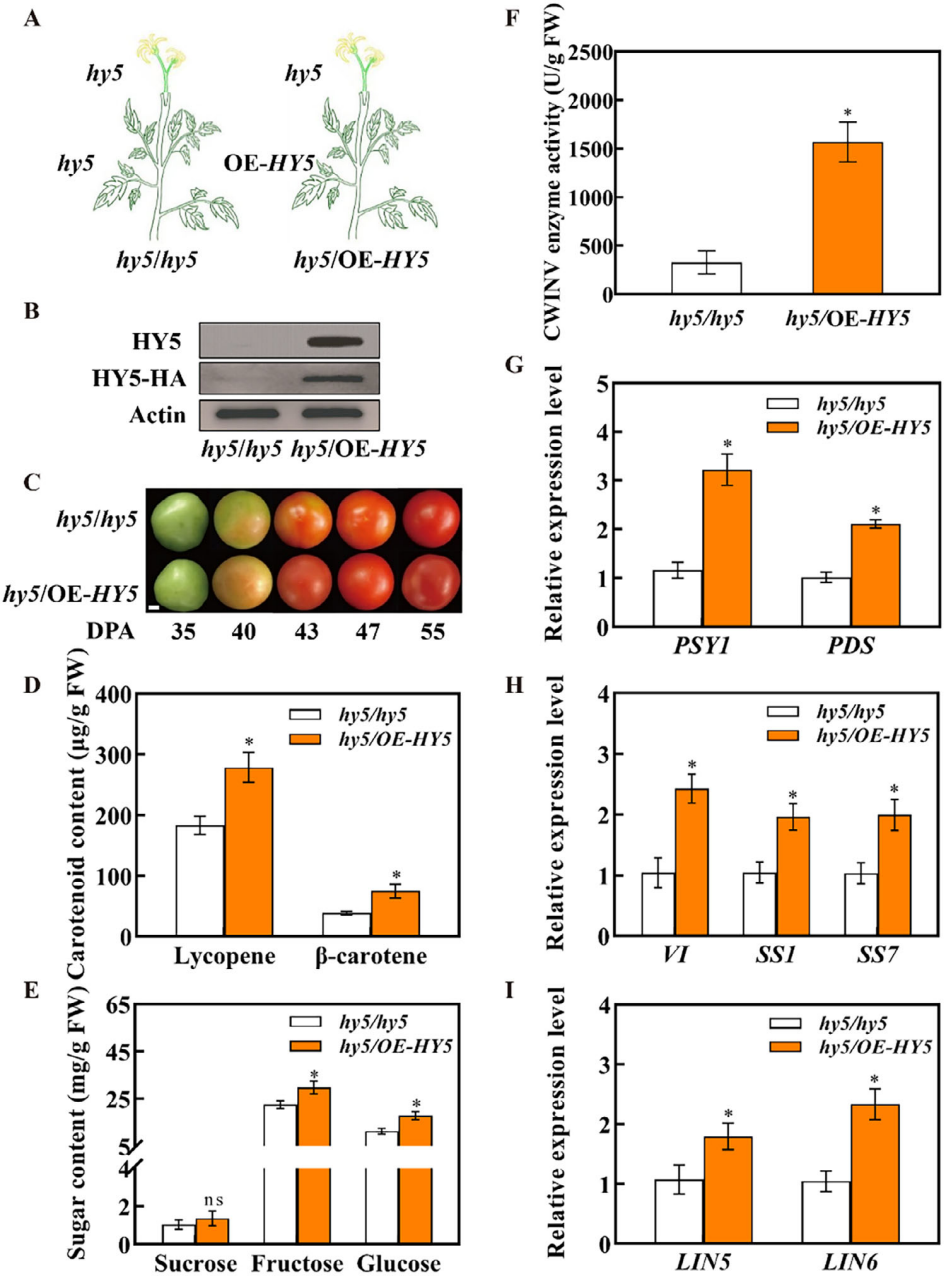
HY5 acts as a systemic signal moving from leaves to fruits. A) Diagram of the grafting experiment, *hy5* and OE‐*HY5* plants were used as plant rootstocks and *hy5* plants were used as fruit scion. B) Immunoblot analysis of the protein abundance of HY5 with anti‐HY5 and anti‐HA antibodies in the fruits from *hy5/hy5* and *hy5*/OE‐*HY5* plants at 35 DPA. C) Fruits collected from *hy5/hy5* and *hy5*/OE‐*HY5* at 35, 40, 43, 47, 55 DPA (Bar = 1 cm). D) Contents of lycopene and β‐carotene in *hy5/hy5* and *hy5*/OE‐*HY5* fruits at 55 DPA. E) Contents of sugars, including sucrose, fructose, and glucose, in *hy5/hy5* and *hy5*/OE‐*HY5* fruits at 55 DPA. F) The activity of CWINV enzyme in *hy5/hy5* and *hy5*/OE‐*HY5* fruits at 55 DPA. G–I) qPCR analysis of carotenoid biosynthetic genes (*PSY1* and *PDS*) and sugar metabolism genes (*VI*, *SS1*, *SS7*, *LIN5* and *LIN6*) in *hy5/hy5* and *hy5*/OE‐*HY5* fruits at 43 DPA. Data are presented as the means of three replicates ± SD; *n* = 3. The asterisks indicate a significant difference according to Student's *t*‐test (*P* < 0.05). qPCR, quantitative PCR.

To understand why the central regulator of light signaling HY5 promoted fruit ripening but bagging did not compromise the accumulation of HY5 protein and impact the progress of fruit ripening, we grafted inflorescence of *hy5* mutant onto *hy5* mutant plants (*hy5*/*hy5*) and OE‐*HY5* plants (*hy5*/OE‐*HY5*) to study whether HY5 protein was involved in the systemic light signaling to regulate fruit ripening (**Figure** **4**A). Using an anti‐HY5 antibody, almost no signs of HY5 protein accumulation were detected in the fruits of *hy5*/*hy5* plants at 40 DPA. In comparison, strong signs of HY5 protein accumulation were detected in the fruits of *hy5*/OE‐*HY5* plants. Notably, the band corresponding to the HY5‐HA fusing protein was also detected in the *hy5* fruits of the *hy5*/OE‐*HY5* plants (Figure 4B). The results suggested that HY5 protein was translocated from the OE‐*HY5* stock to the *hy5* scion fruits, which was consistent with the transcript abundance of HY5 in *hy5*/*hy5* and *hy5*/OE‐*HY5* plants (Figure S6A, Supporting Information). Compared with the *hy5*/*hy5* self‐graft, *hy5* fruits in the *hy5*/OE‐*HY5* graft showed deeper color during fruit ripening (Figure 4C). Remarkably, the expressions of *PSY1*, *PDS*, *ZDS*, and *CrtISO* were increased in the *hy5* fruits from *hy5*/OE‐*HY5* plants at 43 DPA in comparison to fruits from *hy5*/*hy5* self‐graft plants (Figure 4G; Figure S6B, Supporting Information). Accordingly, the lycopene and β‐carotene contents of *hy5*/OE‐*HY5* fruits were higher than those of *hy5*/*hy5* fruits at 55 DPA (Figure 4D). Meanwhile, the contents of fructose and glucose were also increased in the *hy5* fruits of the *hy5*/OE‐*HY5* graft, whereas no differences in sucrose, citric acid, and malic acid were observed between fruits from *hy5*/*hy5* and *hy5*/OE‐*HY5* grafts (Figure 4E; Table S3, Supporting Information). Furthermore, the activity of CWINV exhibited a significant increase in the *hy5* fruits of the *hy5*/OE‐*HY5* graft compared to *hy5*/*hy5* graft (Figure 4F). Consistently, the expressions of sugar metabolism genes (*VI*, *SS1*, *SS7*, *LIN5*, *LIN6*, and *LIN8*) and starch metabolism genes (*BAM8*, *DPE1* and *MEX1*) in *hy5* mutant fruits were enhanced by grafting with OE‐*HY5* at 43 DPA (Figure 4H,I; Figure S6C,D, Supporting Information). Collectively, these results demonstrated the crucial role of HY5 as a signal to improve carotenoid biosynthesis and sugar metabolism by translocating from leaves to fruits in tomato.

### 2.5 Nighttime Supplemental Dim Red and Blue Light is Sufficient to Improve Fruit Quality

**Figure 5:**
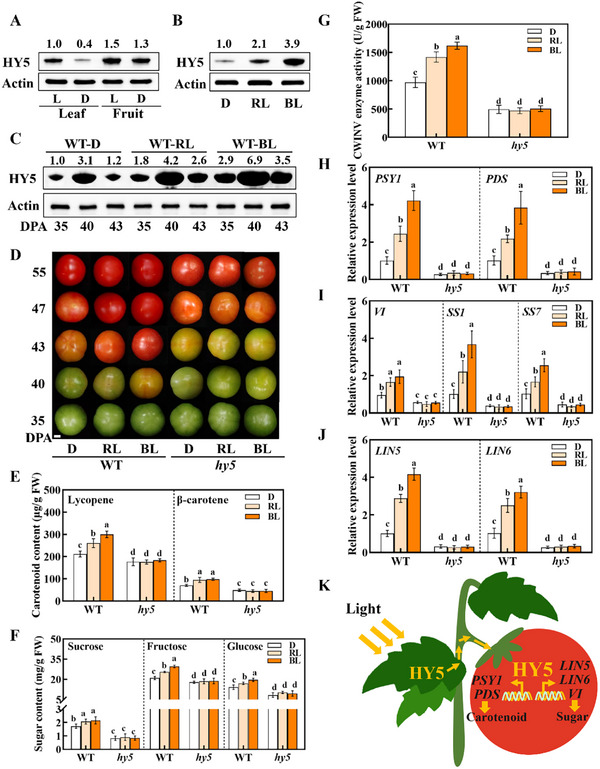
The effect of nighttime supplemental dim red and blue light on tomato fruit quality. A) Immunoblot analysis of HY5 protein abundance from WT leaves and fruits in light (L) and dark (D), respectively. B) Immunoblot analysis of HY5 protein abundance from WT leaves in dark (D), red light (RL), and blue light (BL), respectively. C) Immunoblot analysis of HY5 protein abundance from WT fruits in dark (D), red light (RL), and blue light (BL) at 35, 40, and 43 DPA. D) Fruits collected from WT and *hy5* plants under dark, dim red light, and blue light treatments during fruit ripening (Bar = 1 cm). E) Contents of lycopene and β‐carotene in WT and *hy5* fruits at 55 DPA with dark, dim red light, and blue light treatments. F) Contents of sugars, including sucrose, fructose, and glucose, in WT and *hy5* fruits at 55 DPA with dark, dim red light, and blue light treatments. G) The activity of CWINV enzyme in WT and *hy5* fruits at 55 DPA with different light treatments. H–J) qPCR analysis of carotenoid biosynthetic genes (*PSY1* and *PDS*) and sugar metabolism genes (*VI*, *SS1*, *SS7*, *LIN5*, and *LIN6*) in WT and *hy5* fruits at 55 DPA with different light treatments at night. K) A proposed model that HY5 in the leaves was activated by light and transmitted into the fruits. The mobile and local HY5 in fruits increased the accumulation of carotenoid and sugar by transcriptional activating *PSY1*, *PDS*, *VI*, *SS6*, *SS7*, *LIN5*, and *LIN6*, respectively. Arrows indicated mobile signal and activation. Data are presented as the means of three replicates ± SD; *n* = 3. Different letters indicate a significant difference according to Tukey's test (*P* < 0.05). CWINV, cell wall invertase; qPCR, quantitative PCR.

The short‐day characteristics for the fall and winter months were commonly accompanied by low accumulation of lycopene and sugars in tomato production. As the low intensity of light is enough to induce HY5 accumulation, we then explored the possibility of improving fruit quality with low‐cost lighting at night. To this end, we exposed WT and *hy5* plants with fruits at 30–45 DPA to three light conditions: 8 h white light/16 h dark or 8 h white light plus 6 h red light/10 h dark or 6 h blue light/10 h dark. Red light and blue light were supplied at an intensity of 20 µmol m^−2^ s^−1^. Compared with the less accumulation of HY5 protein in the leaves at night, HY5 accumulation in the fruits during the day is comparable to that at night (**Figure** **5**A). In addition, exposure to red light and blue light during the night not only led to increased HY5 accumulation in the leaves but also in the fruits at night (Figure 5B,C). Importantly, there were significant increases in the accumulation of lycopene, β‐carotene, fructose, and glucose in the fruits and fruit color (Figure 5D–F). Consistently, the CWINV activity and the *PSY1* and *PDS*, *VI*, *SS1, SS7, LIN5*, *LIN6*, and *LIN8* transcripts in the fruits were elevated after the plants were exposed to red light or blue light (Figure 5G–J; Figure S8, Supporting Information). Notably, blue light had more significant effects than red light on the accumulation of HY5 protein, lycopene, β‐carotene, fructose, and glucose and the transcript of *PSY1, PDS*, *VI*, *SS1, SS7, LIN5*, *LIN6*, and *LIN8*. However, such effects were not observed in the fruit of *hy5* plants (Figure 5; Figure S8, Supporting Information).

## 3 Discussion

Tomato fruit ripening is a complex process that is accompanied by intricate metabolic and physiological changes such as chlorophyll breakdown, softening of fruit texture, and the synthesis of carotenoids and sugars. Notably, these processes are regulated not only by hormonal signals but also by the environment. Here we found that the carotenoid and sugar accumulations in the fruits were not completely blocked by bagging. The protein levels of HY5, the critical regulator of light signaling, showed upregulation independent of the local light environment in the bagged fruits during ripening. HY5 positively regulates tomato fruit quality by directly activating the transcription of carotenoid and sugar metabolism genes. Interestingly, the light‐activated HY5 translocated from leaves to fruits and was involved in the systemic regulation of fruit ripening (Figure 5K).

### 3.1 Fruit Quality is Subjected to Local Light and Systemic Regulation

During fruit ripening, metabolic changes occur dramatically, as shown by the increased accumulation of carotenoids and sugars, and the upregulation of genes involved in carotenoid synthesis and starch degradation (Figure 1; Figure S1, Supporting Information). Intriguingly, the accumulation of HY5 protein peaked at the breaker stage, coinciding with the onset of fruit ripening and the highest transcript of genes involved in the synthesis of carotenoids and sugar metabolism. HY5 is activated and stabilized by light signaling.^[^ ^19^ ^]^ However, the timing of fruit color turning and the progress of coloring were unchanged by bagging. Importantly, the induction of HY5 levels at 40 DPA was not disrupted by bagging (Figure 1L), suggesting that the accumulation of HY5 proteins in fruits is independent of local light signaling or is systemically regulated by light‐exposed parts of the plants. Moreover, the coincidence of the induction of HY5 levels and the onset of fruit ripening strongly suggests the contribution of HY5 to ripening in bagged fruits.

In comparison with the control fruits, bagged fruits showed lighter color and less lycopene and β‐carotene contents. The difference relied on the presence of local light illumination on fruits. Light signaling is essential for chloroplast development, while the number and size of chloroplast affect the carotenoid levels in tomato fruits.^[^ ^20^, ^21^, ^22^, ^23^, ^24^ ^]^ Additionally, chloroplast development contributes to photosynthesis and sugar synthesis of fruits.^[^ ^25^, ^26^, ^27^ ^]^ Lack of direct light exposure hindered chloroplast development, leading to reduced levels of carotenoids and sugars in bagged fruits. Taking into account the fact that bagging did not block the carotenoids and sugar accumulation during fruit ripening, it is likely that light signaling dependent and independent are both involved in tomato fruit ripening. Remarkably, this is quite different from the regulation of anthocyanin synthesis, where lack of light exposure as a result of bagging almost completely blocks anthocyanin synthesis in fruits of grapes, strawberries, and pears.^[^ ^28^, ^29^, ^30^ ^]^ HY5 is a critical regulator of anthocyanin synthesis.^[^ ^31^ ^]^ Consequently, carotenoids and anthocyanin synthesis in fruits require different strengths of HY5‐mediated light signaling. Alternatively, the action modes of HY5 in the synthesis of carotenoids and anthocyanin may differ.

### 3.2 HY5 Positively Regulates Tomato Fruit Quality by Directly Activating Genes in Carotenoid Biosynthesis and Sugar Metabolism

Carotenoid synthesis and sugar metabolism during ripening are the critical processes that determine fruit quality. Here, we showed that overexpression of *HY5* promoted the turning of fruit color, whereas knocking out *HY5* delayed color turning (Figure 2A). Consistent with fruit color, lycopene, and β‐carotene contents were higher in OE‐*HY5* fruits but lower in *hy5* mutant fruits as compared with WT fruits. Notably, the differences in fruit color and carotenoid contents between the genotypes were consistently observed in different stages during ripening. Similarly, the contents of TSS and soluble sugar and CWINV activity were increased in OE‐*HY5* fruits but were inhibited in *hy5* mutant fruits during ripening (Figure 2; Figure S5A, Supporting Information). The transcripts of sugar metabolism genes showed consistent changes. Importantly, molecular experiments showed that HY5 directly regulated the transcription of carotenoid synthesis genes *PSY1* and *PDS* and sugar metabolism genes *VI*, *SS1*, *SS7*, *LIN5*, and *LIN6* (Figure 3). Recently, HY5 has been found to activate *SWEET12c* expression by binding directly to the G‐box in its promoter.^[^ ^32^ ^]^ Thus, besides its roles in regulating chloroplast development and subsequent fruit nutritional attributes, HY5 also promotes fruit quality by directly regulating the carotenoid synthesis and sugar metabolism during fruit ripening.

Recent studies demonstrated the role of light signaling in fruit ripening. Red light accelerates fruit color transition, in association with the upregulation of genes encoding ripening‐related regulators and ethylene biosynthesis.^[^ ^33^ ^]^ Indeed, HY5 is required for ethylene biosynthesis and normal fruit ripening in tomato.^[^ ^34^ ^]^ Subsequent ChIP study reveals genes related to ethylene signaling as direct targets of HY5. The finding that the rise of HY5 protein levels coincided with the onset of fruit ripening when ethylene biosynthesis is initiated provides additional evidence that HY5 is a positive regulator of ethylene biosynthesis in tomato fruit ripening. However, blue light inhibits ethylene biosynthesis through HY5‐dependent suppression of *ACS1* in pear.^[^ ^35^ ^]^ Intriguingly, HY5 promotes anthocyanin synthesis in pear,^[^ ^36^ ^]^ whereas ethylene inhibits anthocyanin biosynthesis in pear fruits through ERF105‐dependent activation of *MYB140*, which is a negative regulator of anthocyanin synthesis.^[^ ^37^ ^]^ Although HY5 is critical for the biosynthesis of both carotenoids and anthocyanin, the roles of ethylene in carotenoids and anthocyanin accumulation are contrasting in tomato and pear. This explains the differential HY5‐ethylene interactions in tomato and pear fruits. HY5 interacts with multiple hormones to regulate plant growth and development.^[^ ^13^ ^]^ It will be interesting to study whether HY5 is involved in the regulation of ethylene signaling in tomato fruit ripening.

### 3.3 HY5 is Systemic Signaling in Regulating Fruit Quality with Practical Application

To reveal the role of HY5 in the systemic regulation of fruit quality, we adopted a unique grafting method by grafting the inflorescence of the *hy5* mutant onto the hy5 shoots and OE‐*HY5* shoots, respectively. Intriguingly, the HA‐tagged HY5 proteins were detected in the fruits in *hy5* scion of *hy5*/OE‐*HY5* plants, suggesting that HY5 transports from leaves to fruits to promote fruit ripening (Figure 4B). This is in agreement with our early findings that HY5 protein could translocate from local leaves to systemic leaves, buds, and roots to modify the transcript of *VDE* in photoprotection, *BRC1* in bud outgrowth, *CCD7* in strigolactone biosynthesis and *FER* in Fe^2+^ uptake.^[^ ^38^, ^39^, ^40^, ^41^ ^]^

How to increase the accumulation of pigments and sugars and decrease the accumulation of organic acids is a great challenge to horticulturalists in the production of tomato in the fall and winter. HY5 accumulation in the leaves is highly dependent on light. Results from this study revealed that canopy exposure to dim red or blue light is sufficient to induce the accumulation of HY5, then enhancing the accumulation of carotenoids and sugars (Figure 5). The finding hints that light supplements on leaves are sufficient to promote fruit quality, thus providing an efficient and flexible way to improve fruit quality, especially during the fall and winter seasons.

In conclusion, the results presented in this study demonstrate that HY5 is a positive regulator of tomato fruit ripening by directly regulating the transcription of carotenoid synthesis and sugar metabolism genes. Additionally, HY5 is a mobile signal that moves from leaves to fruits and is involved in the systemic light regulation of fruit ripening (Figure 5K). Our research highlights the significance of HY5 in fruit ripening and provides an effective and flexible strategy for the biofortification of tomato and other vegetables using low‐dose red or blue light, particularly with recent developments in the LED industry.

## 4 Experimental Section

### Plant Materials and Treatments

The wild‐type tomato *Solanum lycopersicum* cv. Ailsa Craig was used in this experiment. Transgenic plants that overexpressed HY5 and the CRISPR‐Cas9‐edited line of HY5 were obtained from previous studies (Wang et al., 2018; Zhang et al., 2020b).^[^ ^42^, ^43^ ^]^ Germinating seeds were placed into 50‐well plates with mixed substrates of peat and vermiculite (2:1, v/v). The seedlings were moved to the pots (height × diameter, 15 cm × 10 cm) with the same mixture at the two‐leaf stage. The plants were cultivated in a controlled growth chamber with the following conditions: 12 h light (photosynthetic photon flux density, 400 µmol m^−2^ s^−1^) at 23 °C and 12 h dark at 20 °C, and with a relative humidity of 70%. The Hoagland nutrient solution was applied to the plants every two days.

The day when the petals of the flowers fully opened was recorded as 0 days post anthesis (DPA). When the fruits grew to approximately 1 cm, they were covered with a paper bag (brown outside and black inside) as the bagging treatment. Mature and ripening fruits were harvested from nine random individual plants simultaneously each day as described previously.^[^ ^44^ ^]^ To determine the effects of supplemental nightly red or blue light on fruit quality in the WT and *hy5* plants, plants were exposed to 8 h photoperiod (9:00–17:00), 8 h photoperiod together with 6 h red light at 660 nm or blue light at 430 nm at an intensity of 20 µmol m^−2^ s^−1^ on the leaves from 17:00.

### Plant Grafting

The seeds of *hy5* and OE‐*HY5* plants were sowed and cultivated in the same manner as previously described. The plants were grafted utilizing the cleft grafting method according to Körpe et al.^[^ ^45^ ^]^ Stems were cut from each genotype with a blade until they bloomed. Shoot scions with flowers from *hy5*, and OE‐*HY5* were reciprocally grafted onto the rootstocks of *hy5* plants. The fully expanded leaves on the scions were removed. The grafted plants were maintained in a dark and moist environment for one week until they could grow normally.

### Chlorophyll and Carotenoids Measurement

Chlorophyll concentrations were determined as previously reported.^[^ ^46^ ^]^ Briefly, frozen fruit samples (0.5 g) were ground into powder and dissolved with 90% acetone. The homogenates were centrifuged at 4 °C for 5 min at 2000 *g*. This procedure was repeated until the precipitate was colorless. The supernatants were collected, and the volume was adjusted to 10 mL. The absorbances of each sample were measured at 663 nm, 645 nm, and 445.5 nm.

Carotenoids measurement was performed as described previously.^[^ ^47^ ^]^ Carotenoids isolated by grinding fruit samples (0.5 g) and then extracting them with trichloromethane: methanol: double distilled water (2:1:1, v/v/v). After centrifugation at 14000 *g* for 10 min at 4 °C, the organic phase supernatants were collected in a new tube. The supernatants were combined and dried with a stream of N_2_. Methanol (350 µL) containing 60% potassium hydroxide was added and derivatized at 60 °C for 30 min in the dark. After adding trichloromethane (700 µL) and double distilled water (350 µL), the samples were extensively vortexed. After centrifugation at 14000 *g* for 10 min at 4 °C, the trichloromethane phase was collected. This procedure was repeated twice, and the organic phases were combined and evaporated to dryness under a stream of N_2_. Ethyl acetate (150 µL) was added to dissolve the precipitate, and the determination was performed by high‐pressure liquid chromatography.

### CWINV Activity Analysis

CWINV activity was detected according to the instructions of a Plant Invertase Activity Detection Kit (BC0130; Solarbio, Beijing, China).^[^ ^48^ ^]^ Briefly, fresh fruit samples (0.1 g) were ground in liquid nitrogen, and an extraction buffer (1 mL) was added. The mixtures were centrifuged at 8000 *g* for 10 min at 4 °C. The supernatants were incubated on ice for further analyses of light absorption at 540 nm.

### Soluble Sugars, Organic Acids and Total Soluble Solids Measurement

The soluble sugars and organic acids were extracted and measured following the description of Zhu et al.^[^ ^47^ ^]^ Fruit sample (0.1 g) was dissolved with 1 mL double‐distilled water (ddH_2_O). After being vortexed for 30 s, the homogenate was incubated at 80 °C for 30 min and followed by the centrifugation at 12000 *g* for 10 min. Another 1 mL ddH_2_O was added to the sediment and the incubation and centrifugation were repeated. The mixture of twice supernatants was diluted by 80% chromatography grade acetonitrile for the determination of soluble sugar and organic acids. The determination was performed by HPLC (Milford, MA, USA), and the standard samples (Solarbio, Beijing, China) were measured at same time for further calculating. The contents of TSS were measured in fruits at 55 DPA with a digital hand‐held refractometer (Atago, Tokyo, Japan).

### RNA‐seq Analysis

Total RNA was extracted from fruits of WT and *hy5* fruits at 40 DPA. Three separate biological replicates were conducted. The mRNA sequencing libraries were constructed, and the sequencing was performed by Novozymes (Beijing, China). The differentially expressed genes were identified by a false discovery rate p‐value ≤ 0.05 and /log_2_foldchange/ ≥ 1. The volcano and KEGG analysis were performed on the platform offered by Novozymes (https://magic‐plus.novogene.com/). The heat map analysis was performed according to log_2_FPKM. The supporting data was listed in the Table S7 (Supporting Information).

### Gene Expression Analysis

Extraction of total RNA from the fruit samples and reverse transcription of the first‐strand cDNA were performed as described by Sang et al.^[^ ^49^ ^]^ The real‐time quantitative reverse transcription PCR was performed on a LightCycler480 detection system (Roche, Basel, Switzerland) using a SYBR Green RT‐PCR Kit (Takara, Shiga, Japan). The relative expression levels of target genes were calculated as described by Livak and Schmittgen,^[^ ^50^ ^]^ and the relative expression of *ACTIN2* and *UBI3* were used as internal controls. The primers shown in Table S4 (Supporting Information) were used for qPCR.

### Protein Extraction and Western Blot

Fruit protein extraction was performed according to the description of Rocco et al.^[^ ^51^ ^]^ Fruit samples (5 g) were ground to powder in liquid nitrogen, followed by the addition of protein extraction buffer (15 mL) and phenol saturated with Tris‐HCl (15 mL) (Sangon Biotech, Shanghai, China). The extracts were vortexed thoroughly, incubated in ice for 15 min, and centrifuged at 12000 *g* for 15 min. The upper aqueous phase was discarded, and the protein extraction buffer was added again for extraction. The supernatants were combined with the five‐fold volume of ammonium acetate methanol (0.1 м) and precipitated at −20 °C for 3–5 h. After centrifugation at 12000 *g* for 30 min, the protein precipitates were recovered and washed with methanol and acetone. Finally, the precipitates were vacuum‐dried to powder. The proteins were separated by 12% SDS‐PAGE and electrophoretically transferred to nitrocellulose membranes (Millipore, Saint‐Quentin, France). A specific HY5 antibody was used to detect the HY5 protein, and an HA antibody was used to detect the HA‐tagged HY5 protein.

### EMSA

The pET‐32a‐His‐HY5 vector was constructed as previously described,^[^ ^52^ ^]^ and the recombinant vector was transferred into *Escherichia coli* strain BL21 (DE3). The His‐HY5 protein was expressed and purified according to the pET purification system (Novagen, Madison, WI, USA). The double‐stranded probes were labeled by Labeling Kit (#89 818, Thermo Fisher Scientific, Waltham, MA, USA). The EMSA was performed with the LightShift Chemiluminescent EMSA Kit (#20 148, Thermo Fisher Scientific, Waltham, MA, USA). The DNA probe sequences shown in Table S5 (Supporting Information) were adopted.

### Dual‐Luciferase Assay

Dual‐luciferase assay was performed as described previously.^[^ ^53^ ^]^ The full‐length coding sequence of *HY5* was constructed using the pGreen II 0029 62‐SK vector, while the promoters of *PDS*, *PSY1*, *VI*, *SS1*, and *SS7* were cloned and ligated to the pGreen II 0800‐LUC vector. These vectors were transformed in *Agrobacterium tumefaciens* strain GV3101. The *A. tumefaciens* strains harboring SK‐HY5 and LUC‐targets vectors were mixed in a 10:1 ratio and infiltrated into *Nicotiana benthamiana* leaves. A double luciferase detection kit (Promega, Madison, WI, USA) was used to measure the ratio of enzyme activity of firefly luciferase (LUC) and renilla luciferase (REN) after 48 h. The relative LUC activity (LUC/REN) of the empty SK vector in combination with the *PDS*, *PSY1*, *VI*, *SS1* and *SS7* promoters was set as one. The primers used were listed in Table S6 (Supporting Information).

### Y1H Assay

Y1H assay was performed according to the method described previously.^[^ ^54^ ^]^ The 300–400 bp sequences containing the HY5‐binding sites in the promoters of *PDS*, *PSY1*, *LIN5*, and *LIN6* were cloned into the pAbAi vector, while the full‐length coding region of *HY5* was ligated to the pGADT7 vector. Y1H assay was performed following the manufacturer's instructions of Matchmatch Gold Yeast One‐Hybrid System (Clontech, Mountain View, CA, USA). The two vectors were co‐transferred into the Y1HGold yeast strain, and an empty AD vector was used as a control. SD/Leu‐ medium with aureobasidin A (50 ng mL^−1^) was used to select the transformed yeast cells. The primers used were listed in Table S6 (Supporting Information).

### Statistical Analysis

A completely randomized design experiment was used. SPSS 19.0 (IBM, Inc., Armonk, NY, USA) was used for statistical analyses, and the differences were determined by Tukey's test or Student's *t*‐test at a level of *P* < 0.05.

## Conflict of Interest

The authors declare no conflict of interest.

## Authors' Contributions

J.Y., Y. Z. conceived and designed the study. J.W., X.L., J.L., and H. D. conducted the experiments. J.W. and X.L. analyzed the data and prepared the first draft; J.H., X.X., J.Y., and Y. Z. contributed to the final editing of the manuscript. All authors have read and approved the final manuscript.

## Supporting information



Supporting Information

Supplementary Table S7

## Data Availability

The data that support the findings of this study are available in the supplementary material of this article.
