# Supplementary material for: Manipulating the Light Systemic Signal HY5 Greatly Improve Fruit Quality in Tomato
Source: Adv Sci (Weinh). 2025 Apr 11;12(23):2500110. doi: 10.1002/advs.202500110 (PMC12199398; doi:10.1002/advs.202500110)
Supplement: Supplementary file 1 — Supporting Information [file ADVS-12-2500110-s002.pdf]

ADVANCED SCIENCE
Open Access

Supporting Information

for *Adv. Sci.*, DOI 10.1002/advs.202500110

Manipulating the Light Systemic Signal HY5 Greatly Improve Fruit Quality in Tomato

Jiachun Wang, Xiaomeng Li, Jiajia Li, Han Dong, Zhangjian Hu, Xiaojian Xia, Jingquan Yu and Yanhong Zhou**

Supporting Information

Manipulating the systemic signaling HY5 greatly improve fruit quality in tomato

Jiachun Wang, Xiaomeng Li, Jiajia, Li, Han Dong, Yanhong Zhou, Xiaojian Xia, Zhangjian Hu, Jingquan Yu

The following supporting data are available for this article:

Figure S1. Transcripts of carotenoid biosynthesis and starch degradation genes during tomato fruit ripening.

Figure S2. The effect of light in carotenoid contents and synthesis genes expression in WT fruits with light and bagging treatment during tomato fruit ripening.

Figure S3. Transcripts of light-regulated and carotenoid biosynthesis genes in WT fruits with light and bagging treatment during tomato fruit ripening.

Figure S4. The transcriptome analysis between WT and *hy5* mutant fruits at 40 DPA in tomato.

Figure S5. The total soluble solids contents and transcripts of *ZDS*, *CrtISO* and *LIN8* in *hy5*, WT and OE-*HY5* fruits at 55 DPA and 43 DPA.

Figure S6. The positions of HY5 *cis*-acting elements on the promoters of *PSY1*, *PDS*, *VI*, *SS1*, *SS7*, *LIN5* and *LIN6*.

Figure S7. Transcripts of HY5 and starch degradation genes in *hy5*/*hy5* and *hy5*/OE-*HY5* fruits.

Figure S8. Transcripts of carotenoid biosynthesis genes (*ZDS* and *CrtISO*) and *LIN8* in WT and *hy5* fruits at 43 DPA with dark, dim red light and blue light treatments.

Table S1. Contents of citric acid and malic acid in WT fruits with light and bagging treatment at 55 DPA.

Table S2. Contents of citric acid and malic acid in the *hy5*, WT and OE-*HY5* fruits at 55 DPA.

Table S3. Contents of citric acid and malic acid in the *hy5*/*hy5* and *hy5*/OE-*HY5* fruits at 55 DPA.

Table S4. List of primer sequences used for qPCR analysis.

Table S5. List of primer sequences used for EMSA assay.

Table S6. List of primer sequences used for vectors constructions.

Table S7. Differentially expressed genes in the fruits of WT and *hy5* mutant at 40 DPA.

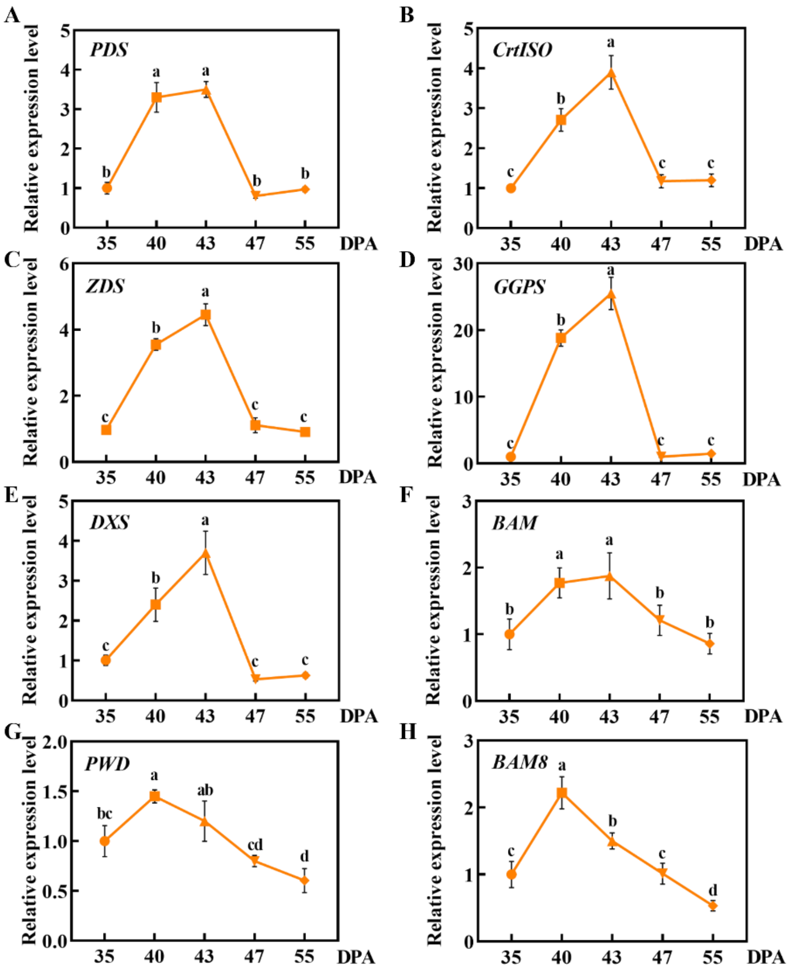

Figure S1. Transcripts of carotenoid biosynthesis and starch degradation genes during tomato fruit ripening. A-H) qPCR analysis of *PDS*, *CrtISO*, *ZDS*, *GGPS*, *DXS*, *BAM*, *PWD* and *BAM8* in WT fruits at 35, 40, 43, 47 and 55 DPA. Data are presented as the means of three replicates ± SD; $n = 3$. Different letters indicate a significant difference according to Tukey's test ($P < 0.05$). DPA, days post anthesis.

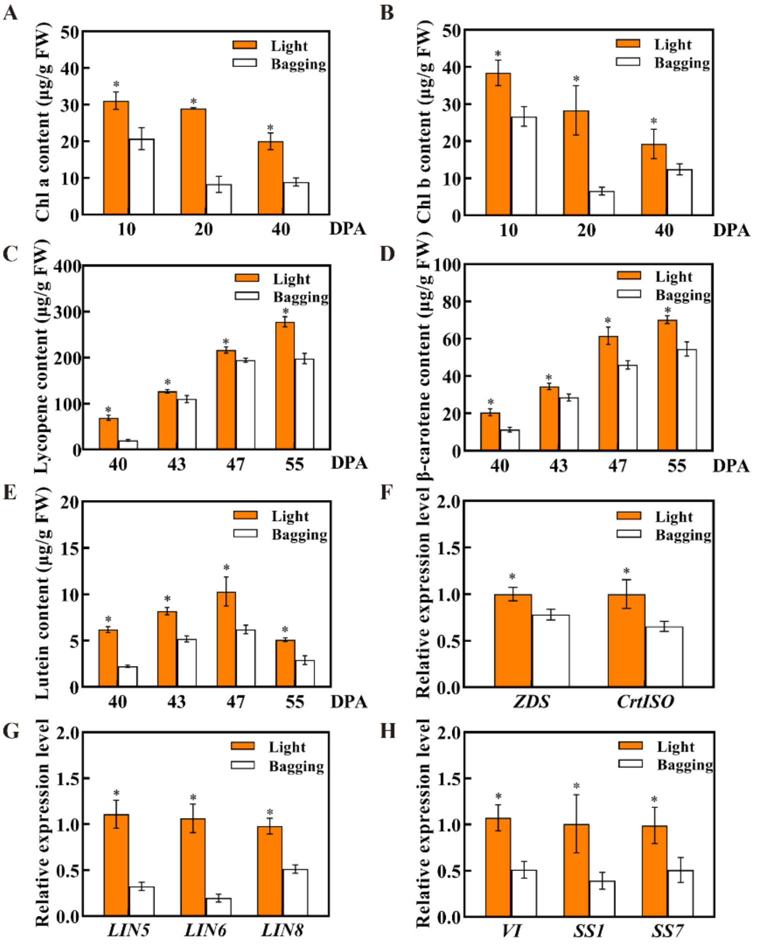

Figure S2. The effect of light in carotenoid contents and synthesis genes expression in WT fruits with light and bagging treatment during tomato fruit ripening. A-B) Contents of Chl a and Chl b in WT fruits with light and bagging treatment at 10, 20 and 40 DPA. C-E) Contents of lycopene, β-carotene and lutein in WT fruits with light ang bagging treatment at 40, 43, 47 and 55 DPA. F-H) qPCR analysis of *ZDS*, *CrtISO*, *LIN5*, *LIN6*, *LIN8*, *VI*, *SS1* and *SS7* in WT fruits with light ang bagging treatment at 43 DPA. All data are presented as the mean values ± SD (n = 3). The asterisks indicate a significant difference, and ns indicates a non-significant difference according to Student's t test (*, $P < 0.05$). WT, wild type; DPA, days post anthesis; SD, standard deviation.

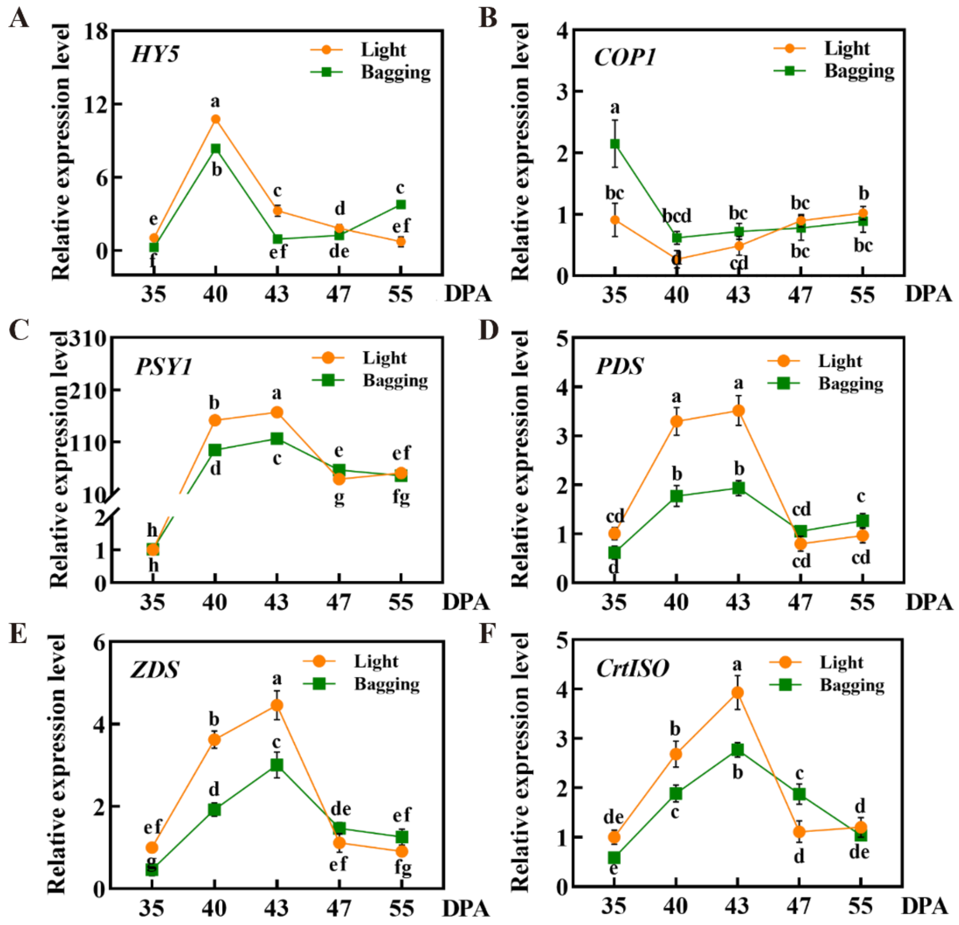

Figure S3. Transcripts of light-regulated and carotenoid biosynthesis genes in WT fruits with light and bagging treatment during tomato fruit ripening. A-B) qPCR analysis of *HY5* and *COP1* in WT fruits with light and bagging treatments at 35, 40, 43, 47 and 55 DPA. C-F) qPCR analysis of *PSY1*, *PDS*, *ZDS* and *CrtISO* in WT fruits with light and bagging treatments at 35, 40, 43, 47 and 55 DPA. All data are presented as the mean values ± SD (n = 3). Different letters indicate significant differences by Tukey's test ($P < 0.05$). WT, wild type; DPA, days post anthesis.

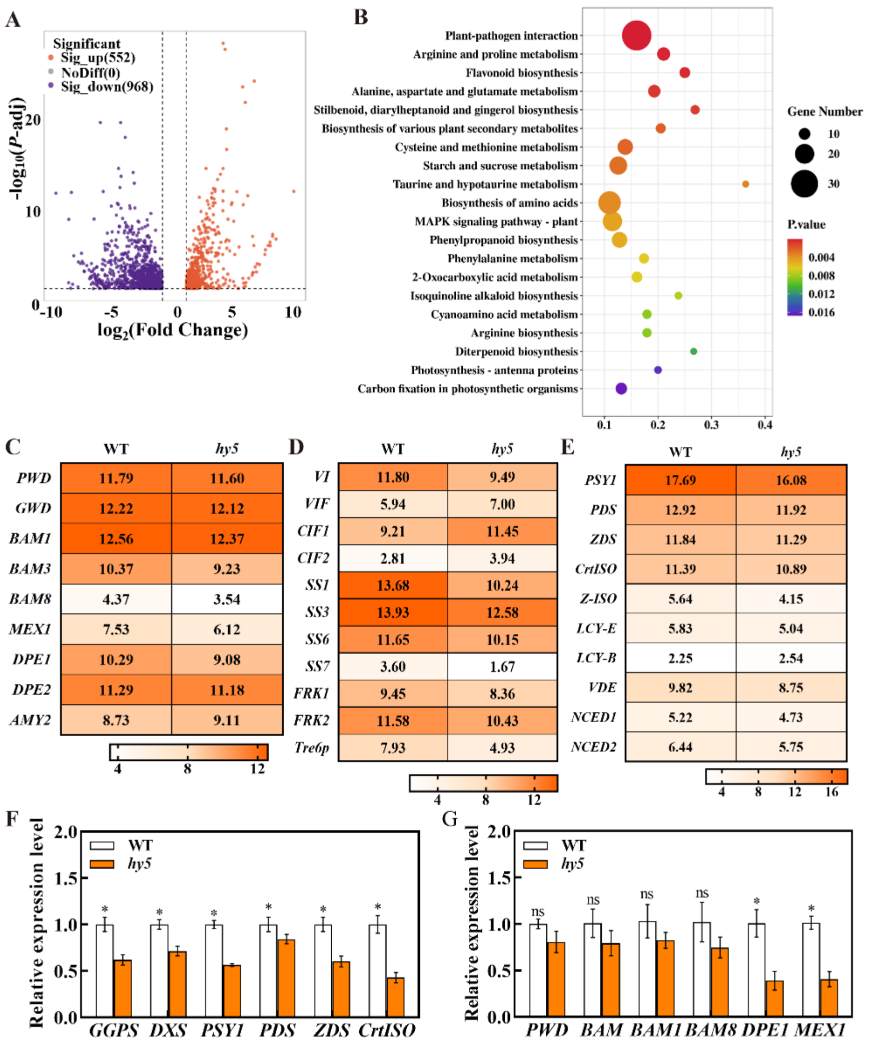

Figure S4. The transcriptome analysis between WT and *hy5* mutant fruits at 40 DPA in tomato. A) Number of differentially expressed genes that upregulated or downregulated in WT fruits compared to *hy5* mutant fruits. B) Scatter plot of the KEGG categories in *hy5* mutant fruits. C-E) Heat map of the differentially expressed starch, sugar and carotenoid metabolism genes identified from an analysis of transcriptome data. The FPKM values were taken as a measure of gene expression, and the scale represents values after normalization of log_2FPKM. F-G) Relative expression of the carotenoid biosynthesis and starch degradation genes in *hy5* mutant and WT fruits at 43 DPA. Asterisks denote a significant difference, and ns indicates a non-significant difference according to Student's *t* test (ns, no significance; *, $P < 0.05$). KEGG, Kyoto encyclopedia of genes and genomes; FPKM, fragments per kilobase of transcript per million mapped reads.

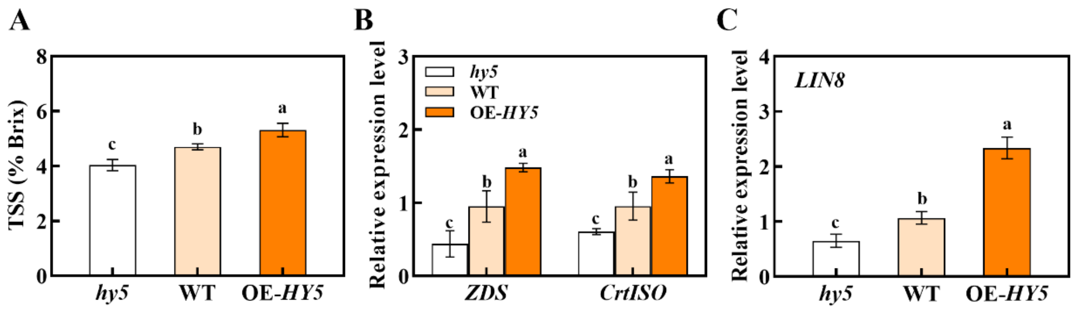

Figure S5. The (A) total soluble solids contents, the transcripts of (B) *ZDS* and *CrtISO*, (C) *LIN8* in *hy5*, WT and OE-*HY5* fruits at 55 and 43 DPA. All data are presented as the mean values ± SD ($n = 8$ in A, $n = 3$ in B and C). Different letters indicate significant differences by Tukey's test ($P < 0.05$). WT, wild type; TSS, total soluble solids.

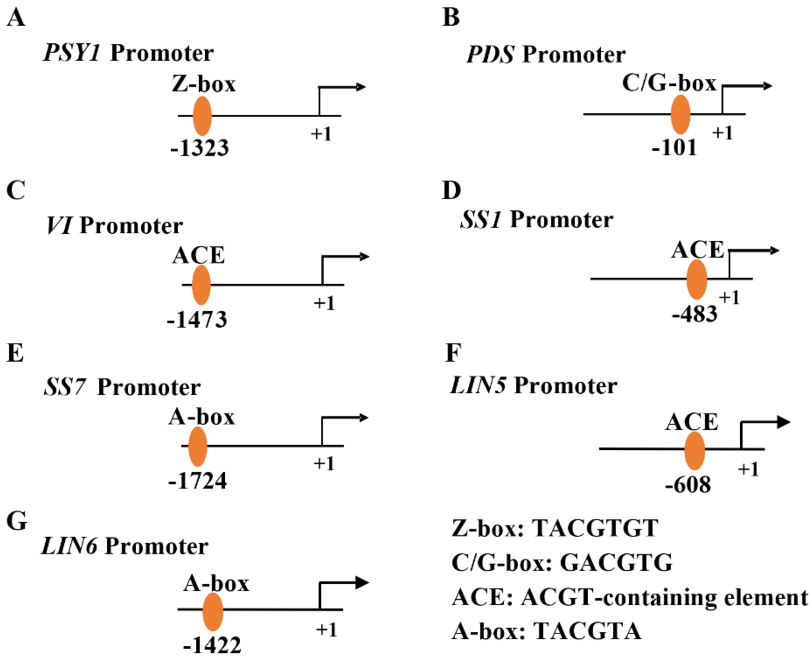

Figure S6. The positions of HY5 *cis*-acting elements on the promoters of *PSY1*, *PDS*, *VI*, *SS6*, *SS7*, *LIN5* and *LIN6*. The orange ellipses indicate the locations of the HY5-acting elements.

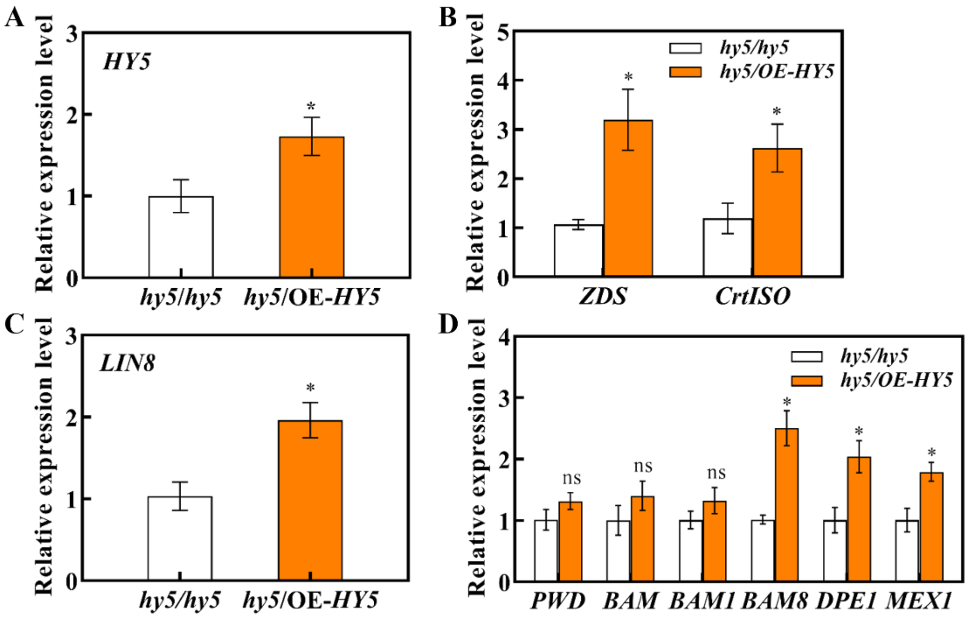

Figure S7. Transcripts of (A) *HY5*, (B) carotenoid biosynthesis genes (*ZDS* and *CrtISO*), (C) *LIN8* and (D) starch degradation genes (*PWD*, *BAM*, *BAM1*, *BAM8*, *DPE1* and *MEX1*) in *hy5*/*hy5* and *hy5*/OE-*HY5* fruits at 43 DPA. All data are presented as the mean values ± SD ($n = 3$). Asterisks denote a significant difference compared with the control group, and ns indicates a non-significant difference according to Student's *t* test (ns, no significance; *, $P < 0.05$).

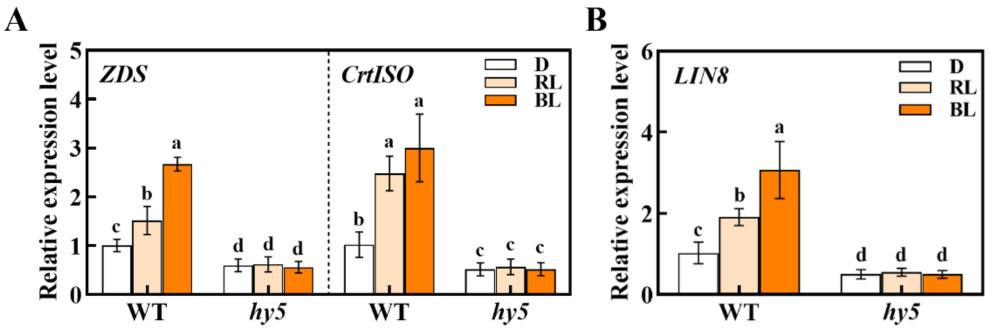

Figure S8. Transcripts of (A) carotenoid biosynthesis genes (*ZDS* and *CrtISO*), (B) *LIN8* in WT and *hy5* fruits at 43 DPA with dark, dim red light and blue light treatments. All data are presented as the mean values ± SD (n = 3). Different letters indicate significant differences by Tukey's test ($P < 0.05$).

Quality attributes	Treatment	
	Light	Bagging
Citric acid [mg/g FW]	5.09±0.51 a	5.67±0.36 a
Malic acid [mg/g FW]	1.25±0.21 a	1.49±0.08 a

Table S1. Contents of citric acid and malic acid in WT fruits with light and bagging treatment at 55 DPA. The data are presented as the mean values ± SD (n = 3). Different letters indicate significant differences (P < 0.05, Tukey's test). SD, standard deviation.

Quality attributes	Tomato lines		
	hy5	**WT**	**OE-*HY5***
Citric acid [mg/g FW]	3.62±0.08 b	5.09±0.51 a	3.06±0.29 b
Malic acid [mg/g FW]	1.57±0.27 a	1.25±0.21 a	0.99±0.40 a

Table S2. Contents of citric acid and malic acid in the *hy5*, WT and OE-*HY5* fruits at 55 DPA. The data are presented as the mean values ± SD (n = 3). Different letters indicate significant differences ($P < 0.05$, Tukey's test). SD, standard deviation.

Quality attributes	Tomato lines	
	hy5*/*hy5	***hy5*/OE-*HY5***
Citric acid [mg/g FW]	2.90±0.24 a	2.68±0.20 a
Malic acid [mg/g FW]	1.02±0.08 a	1.06±0.09 a

Table S3. Contents of citric acid and malic acid in the *hy5*/*hy5* and *hy5*/OE-*HY5* fruits at 55 DPA. The data are presented as the mean values ± SD (n = 3). Different letters indicate significant differences ($P < 0.05$, Tukey's test). SD, standard deviation.

Table S4. List of primer sequences used for qPCR analysis.

Gene	Accession number	Forward primer (5'-3')	Reverse primer (5'-3')
ACTIN2	Solyc11g005330	TGTCCCTATTTACGAGGGTTATGC	CAGTTAAATCACGACCAGCAAGAT
UBI3	Solyc01g056940	GCCGACTACAACATCCAGAAGG	TGCAACACAGCGAGCTTAACC
PSY1	Solyc03g031860	TCTGGAGAACGGACGATGAC	ACTCAACAAGCCCAAATTCCC
PDS	Solyc03g123760	TCATCAACGTTCCGTGCTTC	ATCGGTTTGTGACCAGCATC
ZDS	Solyc01g097810	ACATTGAGGGACAAGGCTCA	CCTCAAGACCTTGGGACGAA
CrtISO	Solyc10g081650	ACTCCTGGGAATGCCTTTCA	CCCTAAGTCAGCTGCAACAC
HY5	Solyc08g061130	TCAGAAGAGTGCCGGAGATG	TTTGAGTCCCAGCTGATGGT
LIN5	Solyc09g010080	AAAGGGATCTCAGCATCACAGG	CGTCTTGGGCATATAGGTCAGC
LIN6	Solyc10g083290	ATCAAGCCCGATAACAATCCA	CCTCACACTCCCAACCAATACTC
LIN8	Solyc10g083300	AAGGATGGGCGGGAATACA	GGCCTGTGCTGGTGTGATT
VI	Solyc03g083910	TCCTTCCCTTTGCAAGACTTGT	TCTCCCTCTTCCCTTTCTTGATG
SS1	Solyc12g009300	CAAGTACCGTAAGATGGCTGAA	TGAAACACTAAACAGAAGCCTCTAC
SS7	Solyc02g081300	TGGCCATTGATGAATAGTTGAAA	GAGGAAGGAATTGCATTCAGAAA

Table S5. List of primer sequences used for EMSA assay.

ID	Primer (5'-3')
EMSA-PSY1-Z-F	GAAATCTACGTGTCTTAA
EMSA-PSY1-Z-R	TTAAGACACGTAGATTTC
EMSA-PSY1-mu-F	GAAAATCTTTTTGTCTTAA
EMSA-PSY1-mu-R	TTAAGACAAAAAGATTTTC
EMSA-PDS-C/G-F	TGGTGGGACGTGTCAAATT
EMSA-PDS-C/G-R	AATTTGACACGTCCCACCA
EMSA-PDS-mu-F	TGGTGGGTTTTGTCAAATT
EMSA-PDS-mu-R	AATTTGACAAAACCCACCA
EMSA-VI-ACE-F	AAGAAAAAAAATGACGTTTTTCCCCTTATT
EMSA- VI-ACE -R	AATAAGGGGAAAAACGTCATTTTTTTTCTT
EMSA-VI -mu-F	AAGAAAAAAAATGAAAATTTTCCCCTTATT
EMSA-VI-mu-R	AATAAGGGGAAAATTTTCATTTTTTTTCTT
EMSA-SS1-ACE-F	CACCCTTTAGACGTATTACAGTAG
EMSA- SS1-ACE-R	CTACTGTAATACGTCTAAAGGGTG
EMSA-SS1-mu-F	CACCCTTTAGAAAAATTACAGTAG
EMSA-SS1-mu-R	CTACTGTAATTTTTCTAAAGGGTG
EMSA-SS7-A-F	GCCCGGTGTCTACGTAAGATTGACAG
EMSA- SS7-A -R	CTGTCAATCTTACGTAGACACCGGGC
EMSA-SS7-mu-F	GCCCGGTGTCTTTTTTAGATTGACAG
EMSA-SS7-mu-R	CTGTCAATCTAAAAAAGACACCGGGC
EMSA-LIN5-ACE-F	GTAAATTATTACGTCAGGCTTTAT
EMSA- LIN5-ACE -R	ATAAAGCCTGACGTAATAATTTAC
EMSA-LIN5 -mu-F	GTAAATTATTTTTTCAGGCTTTAT
EMSA-LIN5-mu-R	ATAAAGCCTGAAAAAATAATTTAC
EMSA-LIN6-A-F	CGCAATTAAATACGTAAGGGCTTTGA
EMSA- LIN6-A -R	TCAAAGCCCTTACGTATTTAATTGCG
EMSA-LIN6-mu-F	CGCAATTAAATTTTTTAGGGCTTTGA
EMSA-LIN6-mu-R	TCAAAGCCCTAAAAAATTTAATTGCG

Table S6. List of primer sequences used for vectors constructions.

Gene	Forward primer (5'-3')	Reverse primer (5'-3')
pET32a-HY5	CGGGATCCATGCAAGAGCAAGCGACGAG	CCCAAGCTTCTACTTCCTCCCTTCCTGTGC
SK-HY5	ATGCAAGAGCAAGCGACGA	CTACTTCCTCCCTTCCTGTGCA
LUC-PSY1	CAAACAAAAATATTAATATGATCGCTATATT	CTACAAGAGGGATCTGGGGAGG
LUC-PDS	GAGAGGACGGCATACTATGTTGTCA	TAAATAAACAAACTAATTTATGATGAAGATGC
LUC-VI	GTCGACACATATCATTAGTTTGGATCT	ACAGGTTATGAAGACAAGAGAAGTAAAAT
LUC-SS1	TTAGACATTTTCAAAAATTTACGTGTAAC	TATAGTTGATTCAGCAGATGGGAAA
LUC-SS7	GTACTCCATCCAATCAGCTAACCA	TGTTGAGATTTTTTTGGGAATTTATAG
LUC-LIN5	GGAATCCACTAAGAAAATCAAAGGTG	GAAGAAGAAAAGAAAGAATATTGGATTATT
LUC-LIN6	ACCATCATAGCAAATGAAACTATACTTATAG	CTTTAATTCTTTCTTTTTGTGTTAAAAAAA
pGADT7-HY5	ATGCAAGAGCAAGCGACGA	CTTCCTCCCTTCCTGTGCACC
pAbAi-*pPSY1*	CCCTAAAATCTAAAATATTAAATATTTGACG	GCTCCACACATTTTGGTCATTTT
pAbAi-*pPDS*	CATCCTGTTCATTTTAATAAACGTACG	CCGAATGCTATAACTAGTAAAAGCGT
pAbAi-*pVI*	GTCGACACATATCATTAGTTTGGATCT	AGGTTATGAAGACAAGAGAAGTAAAATGG
pAbAi-*pSS1*	TTAGACATTTTCAAAAATTTACGTGTAAC	TATAGTTGATTCAGCAGATGGGAAA
pAbAi-*pSS7*	GTACTCCATCCAATCAGCTAACCA	TGTTGAGATTTTTTTGGGAATTTATAG
pAbAi-*pLIN5*	GGAATCCACTAAGAAAATCAAAGGTG	GAAGAAGAAAAGAAAGAATATTGGATTATT
pAbAi-*pLIN6*	ACCATCATAGCAAATGAAACTATACTTATAG	CTTTAATTCTTTCTTTTTGTGTTAAAAAAA
